# Persistent exertional dyspnea following mild COVID-19: potential association with diaphragm impairment

**DOI:** 10.3389/fphys.2026.1968156

**Published:** 2026-10-27

**Authors:** Binaya Regmi, Sitraka Faniry Nantenaina Ratsimba, Benedikt Jörn, Alberto Giannoni, Matthias Boentert, Isabell Pink, Florian Kahles, Michael Dreher, Jens Spiesshoefer

**Affiliations:** 1 Department of Pneumology and Intensive Care Medicine, University Hospital Rheinisch-Westfälische Technische Hochschule (RWTH) Aachen, Aachen, Germany; 2 Interdisciplinary Health Science Centre, Scuola Superiore Sant’Anna, Pisa, Italy; 3 Department of Neurology with Institute for Translational Neurology, University Hospital of Muenster, Muenster, Germany; 4 Department of Medicine, Universitätsklinikum Münster (UKM) Marienhospital Steinfurt, Steinfurt, Germany; 5 Department of Respiratory Medicine and Infectious Diseases, Hannover Medical School, Hannover, Germany; 6 Department of Internal Medicine I-Cardiology, University Hospital Rheinisch-Westfälische Technische Hochschule (RWTH) Aachen, Aachen, Germany

**Keywords:** coronavirus - COVID-19, diaphragm, dyspnea, long COVID, respiratory muscle

## Abstract

**Background:**

Persistent exertional dyspnea is a common symptom in long COVID, and routine cardiopulmonary testing is often unrevealing. The underlying mechanisms remain incompletely understood. Although exertional dyspnea has been linked to diaphragm weakness among hospitalized individuals, studies using state-of-the-art respiratory muscle assessment in non-hospitalized cohorts are lacking.

**Objective:**

This study investigated the role of respiratory muscle function in exertional dyspnea in non-hospitalized patients after mild COVID-19 infection.

**Methods:**

25 outpatients with persistent dyspnea were assessed at a median 24 months after mild COVID-19 infection. Evaluations included pulmonary function testing, 6-minute walk test, echocardiography, twitch transdiaphragmatic pressure (TwPdi) following cervical magnetic stimulation of the phrenic nerve roots, and diaphragm ultrasound. Diaphragm parameters were compared with those of a healthy control group (3:1 matched for age, sex, BMI, and comorbidities).

**Main results:**

Routine blood results, pulmonary function testing or echocardiography showed no clear alternative explanation for dyspnea at the group level. However, COVID-19 individuals exhibited significantly lower diaphragm strength (expressed in TwPdi) than controls (p=0.02), with results varying across dyspnea subgroups (overall ANOVA p-value = 0.009). A negative correlation was observed between TwPdi and dyspnea expressed on mBorg scale (r = -0.67, 95% CI -0.84 to -0.37, p < 0.001). Signs indicating potential structural alterations were observed in post-COVID patients, with significantly reduced diaphragm thickness at functional residual capacity (FRC) and total lung capacity (TLC) compared to controls (p = 0.02 and p < 0.001).

**Conclusions:**

Persistent exertional dyspnea after mild COVID-19 is associated with diaphragm weakness and structural changes despite normal cardiopulmonary findings, supporting a key role for respiratory muscle impairment as a potential contributor to dyspnea in long COVID despite initial mild infection.

## Highlights

- What is already known on this topic
  In hospitalized long-COVID individuals, respiratory muscle impairment is a potential contributor to otherwise unexplained exertional dyspnea. However, studies implementing state-of-the-art methods to assess respiratory muscle function in non-hospitalized populations are still lacking.
- What this study adds
  Even individuals who did not require hospitalization during the acute phase of infection exhibited diaphragm weakness and reduced diaphragm thickness, which were associated with exertional dyspnea.
- How this study might affect research, practice or policy
  In addition to providing a possible pathophysiological explanation for otherwise unexplained exertional dyspnea in long COVID individuals, this identifies a distinct long COVID phenotype and suggests potential for targeted therapeutic interventions.

## Introduction

About one in three or four individuals who have recovered from acute respiratory syndrome coronavirus type 2 (SARS-CoV-2) infection of any severity experience “long COVID” – a heterogeneous condition characterized by a wide range of symptoms and physiological abnormalities that may persist for months or more after recovery from acute illness (Chen et al., 2022). Among the most frequently reported symptoms are fatigue, cognitive dysfunction, exercise intolerance and exertional dyspnea, with the latter representing one of the most functionally limiting symptoms (Wulf Hanson et al., 2022; Singh et al., 2023). Despite its high prevalence and clinical relevance, the pathophysiology of exertional dyspnea in long COVID – including its duration, underlying causes, and degree of specificity - remains poorly understood. Moreover, routine cardiopulmonary evaluations including pulmonary function testing, imaging, and echocardiography frequently fail to identify clinically relevant abnormalities to account for the observed symptom burden (Daher et al., 2020; Durstenfeld et al., 2022).

Several mechanisms have been proposed to explain persistent dyspnea following SARS-CoV-2 infection, including residual parenchymal damage, pulmonary vascular disease, autonomic dysfunction, and impaired peripheral oxygen utilization (Singh et al., 2023). However, in a substantial proportion of patients – particularly those with initially mild disease – these mechanisms do not sufficiently account for the severity of symptoms. This disparity shows that many mechanisms may exist which are not captured by routine cardiopulmonary testing but still could contribute to persistent dyspnea.

Our group previously identified a possible association between diaphragmatic weakness and otherwise unexplained exertional dyspnea as one of the leading symptoms in patients hospitalized with COVID-19, and documented potential improvements after inspiratory muscle strength training (IMT) (Regmi et al., 2023; Spiesshoefer et al., 2024). However, the origin of respiratory muscle strength impairment in previously hospitalized patients might be due to other confounding factors leading to neuropathy or myopathy (e.g. immune mechanisms, pre-existing risk factors, and medications such as steroids or antiviral drugs). For ventilated patients especially, additional factors such as immobilization, extracorporeal membrane oxygenation, and use of paralytics may contribute to the development of respiratory muscle strength impairment (Dres et al., 2017).

With a significant proportion of the population now vaccinated and less virulent SARS-CoV-2 variants prevalent, most individuals presenting with symptomatic long COVID have a history of initial mild acute infection that did not require hospitalization (Ballering et al., 2022; Chen et al., 2022). Even among the non-hospitalized cohort, the symptom burden is similarly high and often remains unexplained by routine clinical or cardiopulmonary assessments (Ballering et al., 2022). In particular, respiratory muscle dysfunction has emerged as a potential contributor and is not captured by standard diagnostic approaches (Polkey, 2023).

To investigate the role of respiratory muscle impairment in unexplained dyspnea in these individuals, this study used state-of-the-art techniques to assess diaphragm muscle strength and function in individuals after mild COVID-19 infection without the necessity of hospitalization. We therefore assessed comprehensive diaphragm strength and function in non-hospitalized individuals with persistent exertional dyspnea after mild COVID-19.

## Methods

### Study design and participants

This prospective non-interventional study (NCT06105450) included outpatients from RWTH University Hospital Aachen who had at least one PCR-positive COVID-19 infection at least a year before assessment and initial mild COVID-19 infection without the need for hospitalization. Those with any comorbidities known to cause exertional dyspnea such as chronic heart failure, COPD, asthma, etc. were excluded. All participants were ambulatory, and none received supplemental oxygen therapy or external respiratory support during or after COVID infection, up to the time of assessment. Furthermore, participants with any medications known to impact respiratory muscle function such as steroids were excluded from the study.

For case-control comparison of diaphragm muscle strength, post-COVID-19 patients were compared with a healthy pre-pandemic control group investigated under identical study conditions using the same technical equipment and study methodology. Controls from pre-pandemic era were selected by frequency matching at an approximate ratio of 3:1 based on age, sex, body mass index, and absence of major comorbidities known to affect respiratory muscle function. Control selection was based on above-mentioned matching variables and was not informed by the present participants’ physiological results.

The study was approved by the local ethics committee (Ethik-Kommission an der Medizinischen Fakultät der RWTH Aachen, EK 23-298) and written informed consent was obtained from all patients. The trial was conducted according to Good Clinical Practice and the Principles of the Declaration of Helsinki 2002.

### Routine follow-up

Patients underwent comprehensive pulmonary function testing, electrocardiography, and transthoracic echocardiography at a median of 24 months after their first SARS-CoV2 infection. Serum, plasma, and whole blood samples were obtained and analyzed as per clinical standards.

Patients answered questionnaires to determine dyspnea (Medical Research Council dyspnea scale (Fletcher et al., 1959), modified Borg dyspnea scale (mBorg) (Borg, 1982), Chronic Respiratory Disease Questionnaire (Guyatt et al., 1987)) and fatigue (modified Fatigue Impact Scale, MFIS (Fisk et al., 1994)), and underwent whole-body plethysmography (MasterLab, Viasys, Hoechberg, Germany) as per current guidelines (Stanojevic et al., 2022). Diffusion capacity was determined after bronchodilation (Graham et al., 2017). Borg dyspnea scale scores were determined before and after a 6-minute walk test (6MWT) without supplemental oxygen to reproduce the dyspnea burden during everyday activities. The classification of patient subgroups was based on exertional dyspnea severity following a 6MWT: no/mild (mBorg dyspnea scale score 0–1); moderate (mBorg dyspnea scale score 2–5); or severe (mBorg dyspnea scale score ≥6) as reported previously (Regmi et al., 2023).

### Invasive inspiratory muscle strength measurements

The experimental setup is demonstrated in **Figures 1A,B**. Twitch transdiaphragmatic pressure (TwPdi) was recorded and analyzed using double balloon catheters (Bösch GmbH, Germany) (Spiesshoefer et al., 2019a). Posterior magnetic phrenic nerve stimulation was used for invasive measurement of twitch transdiaphragmatic pressure (Spiesshoefer et al., 2019a; Regmi et al., 2023)._ENREF_14 After ensuring the correct transnasal placement of esophageal and gastric sensors with few sniff maneuvers, a resting period of a minimum of ten minutes was ascertained to avoid twitch potentiation (Mador et al., 1994). Posterior cervical magnetic stimulation was performed with the subject in the seated position in functional residual capacity (FRC) which was ascertained by requesting participants to hold their breath after a normal, passive exhalation. Magnetic stimuli were delivered using a MagProCompact™ magnetic stimulator equipped with a 2 Tesla 12 cm C-100 circular coil (MagVenture) (Spiesshoefer et al., 2019a; Regmi et al., 2023)_ENREF_14. For posterior cervical magnetic stimulation, the coil was placed at C7 and then moved up toward C6 until the highest reproducible twitch transdiaphragmatic pressure was obtained. Stimulus intensity was increased incrementally until no further increase in TwPdi was observed, and supramaximal stimulation was then used for analysis. Participants were also instructed to repeatedly perform a maximum sniff maneuver and a maximum Mueller maneuver as measures of volitional diaphragm muscle strength to achieve maximum deflection of the transdiaphragmatic pressure curve; the highest of five consecutive efforts was analyzed (Spiesshoefer et al., 2019a; Regmi et al., 2023).

**Figure 1:**
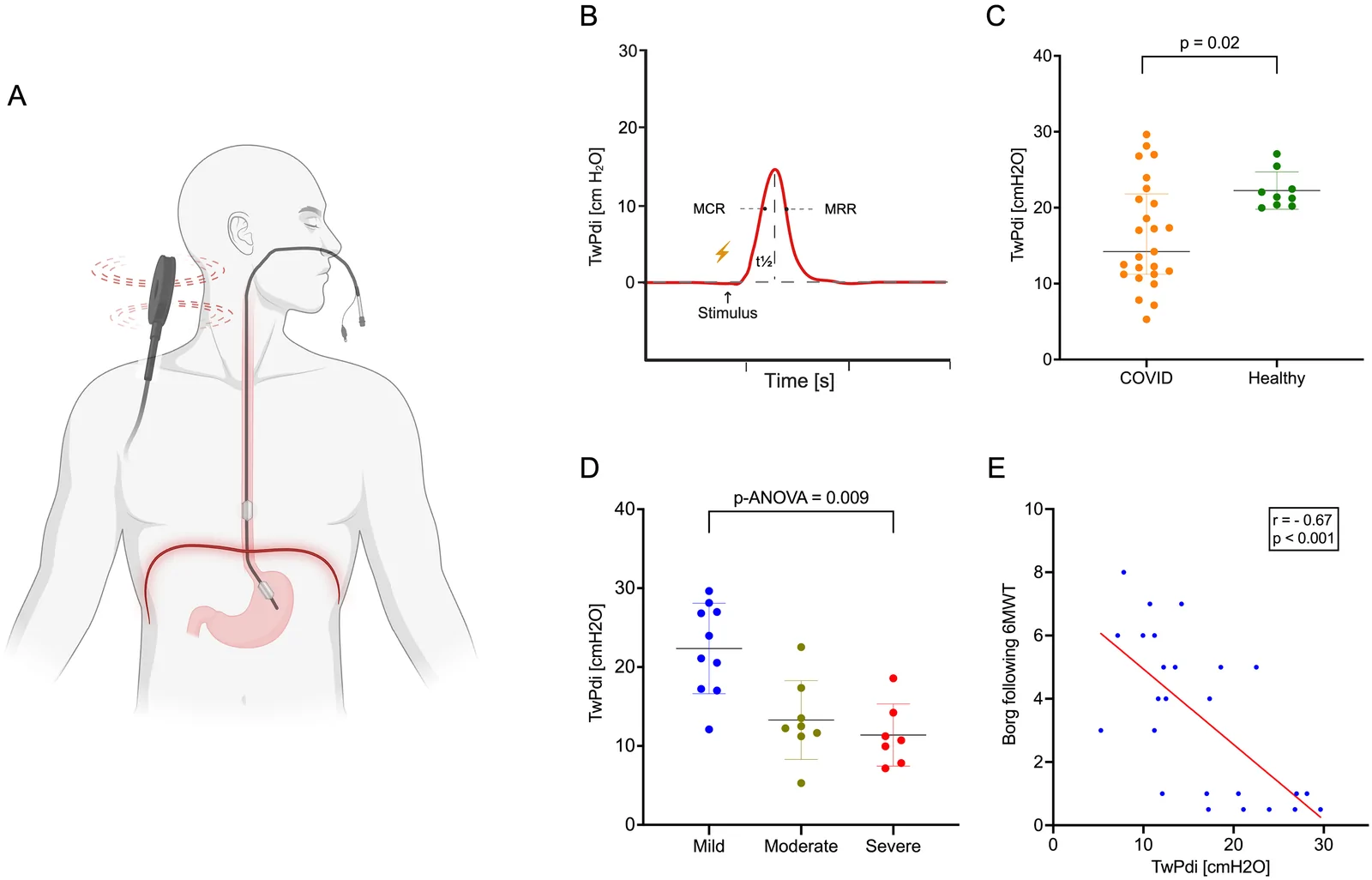
**(A)** Participant in the respiratory physiology laboratory undergoing transnasal insertion of a double balloon catheter to measure esophageal and gastric pressures to derive transdiaphragmatic pressure. A circular magnetic coil for posterior cervical magnetic stimulation (CMS) is positioned. **(B)** Representative twitch transdiaphragmatic pressure (TwPdi) following CMS. Detailed analysis includes pressure amplitude, maximum rate of contraction (MRC) and maximum rate of relaxation (MRR). **(C, D)** Differences in TwPdi in long COVID compared to the healthy cohort, and across dyspnea subgroups. **(E)** Negative correlation between TwPdi and mBorg score following the 6-minute walk test (6MWT). Figure created with biorender.com.

### Twitch superimposition

The DVAI (diaphragm voluntary activation index) reflects the proportion of diaphragmatic muscle mass recruited during voluntary effort, reflecting the degree of voluntary diaphragm activation achieved in a given inspiratory effort. This parameter has been proposed as an indicator of central drive to the diaphragm (Allen et al., 1993). First, maximum TwPdi at FRC and maximum voluntary transdiaphragmatic pressure were obtained, the latter by encouraging the subject to perform a maximum inspiratory effort against an occluded airway at FRC. Subsequently, repetitive increasing stimuli were then delivered during voluntary inspiration while the airway remained occluded. During isovolumetric activation of the diaphragm, twitch interpolation was specifically timed by visual determination of 100% of the individual’s maximum voluntary transdiaphragmatic pressure. Finally, the diaphragmatic voluntary activation index was calculated using the formula (Allen et al., 1993):


$$DVAI=1-\frac{TwPdi\;at\;any\;given\;inspiratory\;effort}{max.\;TwPdi\;at\;FRC\;}\;x\;100$$

### Diaphragm ultrasound

Diaphragm ultrasound was performed on the right hemidiaphragm as previously described (Spiesshoefer et al., 2020). The diaphragm was visualized using a 10-MHz probe placed in the posterior axillary line between the 8^th^ and 10^th^ intercostal spaces and was identified with the characteristic three-layered structure. Measurements were first obtained at total lung capacity (TLC), then repeated at the same location after exhalation to FRC. All measurements were performed three times and the average value for each parameter was calculated at each lung volume. The diaphragm thickening ratio (DTR) was calculated as thickness at TLC divided by thickness at FRC (Spiesshoefer et al., 2020).

### Statistical analysis

Statistical analyses were performed using GraphPad Prism software (Version 11.0, GraphPad Software, San Diego, USA). Data were expressed as median (interquartile range [IQR]). The Shapiro-Wilk test was used for normality testing. For between-group comparisons, unpaired t-tests were used for normally distributed data. For non-normal distribution, Mann-Whitney U test was used. For dyspnea severity subgroup comparisons, one-way ANOVA with Tukey *post-hoc* test for significant differences was used for normally distributed data. Otherwise, the Kruskal-Wallis test with Dunn’s *post-hoc* test (Bonferroni correction) was used. Pearson correlation coefficient was calculated for correlation analyses as the reported correlation parameters passed the normality testing. *P* ≤ 0.05 was considered statistically significant. To improve interpretability, 95% confidence intervals (CI) were additionally reported for the principal effect estimates and the main correlation analysis.

The primary endpoint was TwPdi following cervical magnetic stimulation. Secondary measures included additional invasive respiratory muscle parameters, diaphragm ultrasound metrics, and questionnaire-derived symptom measures. Subgroup analyses according to dyspnea severity and analyses of secondary endpoints were considered exploratory.

Because dyspnea subgroups were defined using the post-6MWT mBorg score, the subgroup comparison and the continuous correlation analysis represent categorical and continuous descriptions of the same symptom variable rather than independent confirmatory analyses.

## Results

### Study population

Twenty-five patients with persistent exertional dyspnea were analyzed at a median of 24 months [IQR 23–29] following initial SARS-CoV-2 infection. Baseline demographic and clinical characteristics of the cohort are summarized in **Table 1**. No relevant comorbidities that could confound respiratory muscle function were identified. In addition, routine cardiopulmonary evaluation, including pulmonary function testing, capillary blood gas analysis or transthoracic echocardiography, revealed no major clinically significant abnormalities, although mild abnormalities on capillary partial pressure of oxygen (pO2) and DLCO were present in some participants (**Table 2**). Specifically, DLCO was below the lower limit of normal (LLN) in nine participants, although none had a DLCO <60% predicted. At the time of assessment, only one of the participants was a current smoker, with four others being former smokers.

**Table 1:** Demographics, Dyspnea and respiratory muscle strength at median 24 months after first COVID-19 infection compared with controls and in subgroups based on the severity of exertional dyspnea.

Variables		Comparison COVID-19 vs Controls			COVID-19 subgroups according to dyspnea severity			
	All non-hospitalized post-COVID-19 individuals (n=25)	Controls (n=9)	Mean difference (95% CI)	*p-*value	Severe (n=7)	Moderate (n=8)	No/mild (n=10)	*p-*value across subgroups
Demographics								
Male sex, %	10 (40)	4 (44)	–	0.82	2 (29)	2 (25)	6 (60)	0.27
Age, year	46.0 (40.0; 55.5)	45.0 (38.5; 57.0)	-2.2 (-12.4; 8.1)	0.73	52.0 (46.0; 61.0)	41.0 (39.5; 46.5)	43.5 (33.5; 53.8)	0.29
Height, m	1.72 (1.68; 1.81)	1.74 (1.68; 1.79)	0.02 (-0.03; 0.07)	0.82	1.68 (1.64; 1.72)	1.73 (1.69; 1.79)	1.73 (1.70; 1.82)	0.26
Weight, kg	74.0 (66.5; 94.0)	70.0 (65.0; 82.5)	7.9 (-1.4; 16.5)	0.39	100.0 (64.0; 106.0)	73.5 (69.2; 88.0)	72.5 (65.0; 84.2)	0.28
BMI, kg/m^2^	24.1 (22.9; 34.1)	24.0 (22.6; 25.6)	2.9 (0.2; 5.9)	0.40	27.5 (23.8; 35.8)	23.7 (22.9; 32.1)	24.3 (22.4; 30.4)	0.35
Time since first infection, months	24.0 (23.0; 29.0)	–	–	–	25.0 (23.0; 29.0)	24.0 (23.0; 30.0)	20.0 (20.0; 26.0)	0.30
Dyspnea								
mMRC dyspnea scale score	2.0 (1.0; 3.0)	–	–	–	3.0 (3.0; 3.0)	2.0 (2.0; 3.0)	1.0 (1.0; 1.0)	**<0.001†§‡**
mBorg dyspnea scale score	4.0 (1.0; 5.5)	–	–	–	6.0 (6.0; 7.0)	4.0 (3.2; 5.0)	0.7 (0.5; 1.0)	**<0.001†§‡**
MFIS score	17.0 (5.0; 33.5)	–	–	–	34.0 (17.0; 62.0)	23.5 (17.7; 50.2)	4.5 (0.7; 5.7)	**<0.001§‡**
CRQ dyspnea domain score	4.3 (3.4; 6.4)	–	–	–	3.4 (2.7; 3.4)	3.7 (3.3; 4.6)	6.4 (5.1; 7.0)	**<0.001§‡**
CRQ fatigue domain score	4.7 (3.5; 5.4)	–	–	–	4.0 (2.5; 5.0)	4.9 (3.3; 5.2)	5.5 (3.9; 5.9)	0.07
CRQ emotional function score	5.0 (4.1; 5.9)	–	–	–	4.6 (3.3; 6.0)	4.7 (3.8; 5.2)	5.7 (4.7; 6.3)	0.08
CRQ mastery score	6.0 (5.2; 7.0)	–	–	–	5.5 (2.7; 6.0)	5.2 (3.7; 6.5)	7.0 (6.6; 7.0)	**0.002§‡**
Non-volitional invasive RMS								
CMS TwPdi, cmH_2_O	14.2 (11.2; 21.8)	21.4 (20.3; 24.0)	-5.9 (-9.3; -2.47)	**0.02**	10.7 (7.8; 14.2)	12.4 (11.3; 16.4)	22.5 (17.2; 27.3)	**0.009 §‡**
CMS twPes, cmH_2_O	–8.2 (–13.6; –4.7)	–12.9 (–15.7; –11.0)	2.9 (-0.3; 6.1)	0.17	–6.2 (–11.1; –4.0)	–5.3 (–9.1; –3.6)	–15.9 (–20.7; –8.5)	**0.002§‡**
CMS twPgas, cmH_2_O	6.1 (3.4; 7.9)	5.4 (2.4; 8.6)	-0.1 (-3.2; 3.0)	0.83	4.7 (2.7; 5.6)	7.3 (5.2; 9.0)	6.8 (3.4; 9.2)	0.20
CMS MRR normalized, cmH_2_O/msec	–10.6 (–20.6; –7.5)	–7.1 (–15.3; –5.3)	-12.0 (-27.7; 3.7)	0.31	–11.3 (–63.3; –8.1)	–13.1 (–21.1; –6.7)	–8.3 (–15.2; –6.9)	0.47
CMS MCR normalized, cmH_2_O/msec	25.7 (19.0; 36.3)	28.3 (21.6; 34.9)	5.7 (-4.7; 16.1)	0.91	32.3 (13.6; 47.2)	26.0 (19.9; 34.7)	23.1 (18.5; 37.7)	0.82
Volitional invasive RMS								
Sniff Pdi, cmH_2_O	85.2 (56.9; 96.7)	84.2 (66.9; 110.8)	-4.7 (-28.3; 18.9)	0.60	57.8 (50.1; 77.9)	70.8 (51.8; 93.0)	96.7 (87.2; 100.7)	**0.03§**
Sniff Pes, cmH_2_O	–54.4 (–66.5; –31.9)	–71.0 (–84.6; –43.6)	3.3 (-42.6; 19.3)	0.72	–41.8 (–56.7; –2.7)	–38.8 (–61.5; –29.5)	–66.4 (–90.9; –51.9)	**0.02§**
Mueller Pdi, cmH_2_O	70.8 (57.4; 99.9)	83.6 (56.1; 110.1)	-4.1 (-31.4; 23.2)	0.76	75.2 (63.2; 88.4)	62.0 (39.8; 78.5)	103.6 (58.7; 126.3)	**0.05‡**
Mueller Pes, cmH_2_O	–51.6 (–65.0; –40.1)	–45.8 (–94.0; –36.2)	-1.9 (-29.8; 26.0)	0.84	–54.4 (–69.0; –38.3)	–40.2 (–52.5; –25.2)	–52.4 (–85.0; –43.0)	0.07
Valsalva Pgas, cmH_2_O	103.1 (69.0; 141.2)	88.0 (54.6; 131.3)	22.5 (-37.2; 82.2)	0.56	76.0 (69.0;128.8)	74.7 (50.5; 118.1)	109.3 (103.2; 214.0)	0.09
Cough Pgas, cmH_2_O	114.4 (87.80; 152.9)	121.7 (97.2; 155.1)	8.6 (-26.7; 43.8)	0.95	111.7 (79.4; 144.8)	89.1 (83.5; 135.6)	133.5 (102.9; 171.6)	0.14
Neural control								
DVAI, %	67.9 (48.2;86.3)	80.0 (71.3; 88.8)	-10.2 (-23.2; 2.7)	0.21	65.0 (53.0;83.0)	61.8 (36.2;88.1)	72.0 (43.8;90.0)	0.75
Diaphragm ultrasound								
Thickness at FRC, cm	0.14 (0.11;0.18)	0.20 (0.14; 0.26)	-0.06 (-0.11; -0.01)	**0.02**	0.15 (0.12;0.18)	0.12 (0.10;0.17)	0.16 (0.12;0.22)	0.23
Thickness at TLC, cm	0.31 (0.25;0.36)	0.50 (0.38; 0.68)	-0.19 (-0.36; -0.03)	**<0.001**	0.28 (0.25; 0.33)	0.25 (0.23;0.33)	0.35 (0.32;0.49)	**0.02‡**
DTR	2.2 (1.8; 2.7)	2.8 (2.0; 3.1)	-0.3 (-0.8; 0.2)	0.07	1.9 (1.8;2.7)	2.3 (1.8;2.7)	2.3 (2.1;2.6)	0.63
**Sniff nasal pressure, cmH_2_O**	80.0 (67.0; 96.9)	–	–	–	75.2 (67.3; 125.9)	71.2 (53.9; 85.3)	94.5 (80.0; 115.1)	0.21
Sniff nasal pressure, % predicted	89.0 (64.9; 105.2)	–	–	–	90.3 (66.0; 129.0)	77.6 (61.3; 88.1)	101.7 (70.6; 119.3)	0.27

Values are median (interquartile range).

†Significant differences (*P <* 0.05) between moderate and severe dyspnea subgroups if p-value across subgroups is significantly different (*P <* 0.05).

‡Significant differences (*P <* 0.05) between mild and moderate dyspnea subgroups if p-value across subgroups is significantly different (*P <* 0.05).

§Significant differences (*P <* 0.05) between mild and severe dyspnea subgroups if p-value across subgroups is significantly different (*P <* 0.05).

CI; confidence interval; mMRC, Modified Medical Research Council; MFIS, Modified Fatigue Impact Scale; CRQ, Chronic Respiratory Disease Questionnaire; RMS, respiratory muscle strength; CMS, cervical magnetic stimulation (of the phrenic nerve roots); tw, twitch; Pdi, transdiaphragmatic pressure; Pes, esophageal pressure; Pgas, gastric pressure; MRR, maximum relaxation rate; MCR, maximum contraction rate; DVAI, diaphragm voluntary activation index; FRC, functional residual capacity; TLC, total lung capacity.

The MFIS score assesses the impact of fatigue on a patient’s daily life. A higher score indicates that the patient is more likely to be experiencing significant fatigue (range 0-84).

The CRQ scores evaluate the limitations caused by chronic lung diseases across four domains: dyspnea, fatigue, emotion, and mastery. A smaller score suggests that the patient is experiencing greater limitations in each domain, with a range from 1 (indicating maximum impairment) to 7 (indicating no impairment).

Statistically significant p-values (p < 0.05) are shown in bold.

**Table 2:** Pulmonary function tests, 6-minute walk test, echocardiography and laboratory findings for the overall study population, and in patient subgroups based on the severity of exertional dyspnea.

Variables	All non-hospitalized COVID-19 individuals (n=25)	Exertional dyspnea severity subgroup				Normal values, if applicable
		Severe (n=7)	Moderate (n=8)	No/mild (n=10)	*p-*value across subgroups	
Diffusion parameters and capillary blood gases						
DLCO, % predicted	71.0 (66.5; 88.5)	71.0 (62.0; 76.5)	75.5 (67.5; 95.0)	74.5 (62.7; 87.2)	0.52	
DLCO/VA, % predicted	83.0 (75.0; 98.5)	83.0 (76.0; 113.0)	90.0 (75.5; 97.7)	78.0 (66.5; 106.2)	0.87	
Capillary PO_2_, mmHg	79.6 (72.2; 90.6)	73.4 (64.8; 76.3)	89.4 (70.0; 92.1)	84.9 (79.0; 91.9)	**0.03**	
Capillary PCO_2_, mmHg	37.4 (35.0; 38.9)	36.2 (33.1; 39.5)	36.7 (35.0; 38.1)	37.6 (36.5; 40.6)	0.63	
pH	7.43 (7.41; 7.45)	7.43 (7.41; 7.46)	7.42 (7.41; 7.47)	7.43 (7.43; 7.44)	0.83	
Base excess, mmol/L	0.6 (-0.2; 2.2)	1.3 (-0.5; 2.7)	0.1 (-0.4; 2.4)	0.9 (0.6; 2.0)	0.71	
Pulmonary function parameters						
TLC, % predicted	110.0 (98.5; 120.8)	105.0 (87.0; 111.0)	119.5 (98.2; 120.9)	108.0 (96.2; 129.2)	0.39	
Vital capacity, % predicted	102.5 (93.0; 111.7)	102.0 (82.0; 107.0)	106.5 (90.5; 116.5)	107.4 (98.0; 111.0)	0.28	
RV/TLC, % predicted	107.0 (100.0; 116.5)	111.0 (100.0; 115.0)	108.3 (102.7; 136.2)	105.0 (91.2; 119.5)	0.45	
FEV_1_, % predicted	88.5 (76.0; 101.3)	81.0 (69.0; 100.0)	87.5 (74.0; 100.5)	94.0 (86.0; 106.0)	0.36	
FEV_1_/FVC, %	80.0 (71.4; 87.3)	79.3 (70.0; 83.2)	78.5 (68.2; 87.3)	86.7 (79.5; 108.0)	0.06	
6MWT						
6MWD, m	515 (420; 610)	440 (400; 515)	560 (485; 595)	625 (420; 640)	0.15	
6MWD, % predicted	79.5 (69.4; 89.4)	81.2 (72.5; 84.9)	77.5 (69.1; 90.0)	75.3 (62.7; 115.7)	0.82	
Heart rate before, bpm	80.0 (67.8; 90.8)	80.0 (72.0; 83.5)	87.0 (72.5; 92.5)	75.0 (62.0; 79.0)	0.55	
Heart rate after, bpm	99.5 (84.3; 113.5)	104.0 (92.5; 109.0)	106.0 (84.5; 120.0)	85.0 (81.0; 89.5)	0.39	
SpO2 before, %	98.0 (97.0; 98.0)	98.0 (97.5; 98.0)	98.0 (98.0; 98.5)	98.0 (96.0; 98.0)	0.58	
SpO2 after, %	97.0 (97.0; 98.0)	98.0 (97.5; 98.5)	97.0 (97.0; 98.0)	97.0 (97.0; 99.0)	0.90	
Echocardiography						
LVEF, %	55.0 (55.0; 60.5)	55.0 (55.0; 63.0)	55.0 (55.0; 61.5)	55.0 (54.75; 60.25)	0.97	
TAPSE, mm	2.3 (2.1; 2.5)	2.3 (2.2; 2.4)	2.4 (2.2; 2.7)	2.1 (1.9; 2.5)	0.14	
Hematology						
White blood cells, 1/nL	6.8 (5.5; 8.0)	7.0 (4.9; 7.3)	6.4 (5.2; 8.9)	6.7 (5.8; 14.0)	0.29	4–10
Hemoglobin, g/dL	14.4 (13.5; 15.1)	14.4 (13.4; 15.4)	14.1 (13.5; 14.7)	14.0 (13.6; 15.6)	0.73	11.2–15.7
Platelets, 1/nL	259.0 (205.0; 308.0)	259.0 (180.0; 330.0)	271.5 (208.0; 300.7)	237.5 (210.5; 326.5)	0.96	150–400
Clinical chemistry						
Bilirubin-direct, mg/dL	0.4 (0.3; 0.6)	0.4 (0.3; 0.5)	0.4 (0.2; 0.5)	0.6 (0.3; 0.6)	0.37	<1.2
ALT, U/L	23.5 (18.0; 35.2)	30.0 (17.0; 53.0)	21.0 (18.0; 24.0)	25.0 (19.2; 61.5)	0.22	<35
NT-proBNP, pg/mL	80.8 (51.4; 146.5)	103.0 (28.5; 150.0)	65.5 (40.2; 142.0)	84.5 (77.2; 161.0)	0.80	<486
Creatinine, mg/dL	0.8 (0.7; 0.9)	0.8 (0.7; 0.9)	0.7 (0.7; 0.8)	0.8 (0.8; 1.0)	0.47	<1.0
C-reactive protein, mg/L	1.2 (0.6; 3.3)	1.9 (0.9; 4.7)	1.0 (0.6; 3.4)	1.2 (0.6; 2.7)	0.23	<5

Values are median (interquartile range).

6MWT, 6-minute walk test; 6MWD, 6-minute walk distance; ALT, alanine aminotransferase;

†Significant differences (*P<*0.05) between moderate and severe dyspnea groups, if p-value across subgroups is significantly different (*P<*0.05).

‡Significant differences (*P<*0.05) between mild and moderate dyspnea groups, if p-value across subgroups is significantly different (*P<*0.05).

§Significant differences (*P<*0.05) between mild and severe dyspnea groups, if p-value across subgroups is significantly different (*P<*0.05).

DLCO, diffusing capacity for carbon monoxide; VA, alveolar volume; PO_2,_ partial pressure of oxygen; PCO_2_, partial pressure of carbon dioxide; pH, potential of hydrogen; TLC, total lung capacity; RV, residual volume; FEV_1,_ forced expiratory volume in 1 second; FVC, forced vital capacity; 6MWT, 6-minute walk test; 6MWD, 6-minute walk distance; LVEF, left ventricular ejection fraction; TAPSE, tricuspid annular plane systolic excursion; ALT, alanine aminotransferase; NT-proBNP, N-terminal pro B–type natriuretic peptide.

Statistically significant p-values (p < 0.05) are shown in bold.

Eleven patients had experienced a single SARS-CoV-2 infection, while the remaining 14 had experienced two infections. At enrollment, 96% of patients had received at least two doses of an mRNA-based SARS-CoV-2 vaccine.

### Dyspnea and fatigue

At follow-up, exertional dyspnea severity was reported as no/mild in 10 patients (40%), moderate in 8 patients (32%), and severe in 7 patients (28%). Patients with increasing exertional dyspnea reported significantly higher scores on the mMRC and mBorg scales, indicating greater perceived breathlessness across subgroups (**Table 1**).

Fatigue burden was also prominent within the cohort. Correlation analysis demonstrated a significant association between dyspnea severity and fatigue, with both mBorg dyspnea scores following the 6MWT and mMRC scores correlating positively with MFIS score (r = 0.94/0.75, both p < 0.001). During the 6MWT, no relevant exertional desaturation was observed at the group level (**Table 2**).

### Respiratory muscle strength

Non-volitional diaphragmatic strength measurements indicated evidence of diaphragm impairment among post-COVID individuals: TwPdi following posterior cervical magnetic stimulation was significantly lower in post-COVID-19 patients when compared with controls (mean difference -5.9 cmH_2_O, 95% CI -9.3 to -2.5, p = 0.02) (**Table 1**; **Figure 1C**). Thirteen patients (52%) had TwPdi values below the lower limit of normal (LLN; <16 cmH2O) based on previously published data (Spiesshoefer et al., 2019a).

When stratified by dyspnea severity, a significant and progressive reduction in TwPdi was observed with increasing dyspnea severity (overall ANOVA p-value = 0.009) (**Table 1**; **Figure 1D**). Furthermore, we observed a significant inverse correlation between TwPdi and exertional dyspnea severity expressed in mBorg scale following 6MWT (r = –0.67, 95% CI -0.84 to -0.37, p<0.001) (**Figure 1E**). In contrast, invasive volitional diaphragmatic pressure measurements were similar in patients and controls (**Table 1**). These findings do not suggest a major group-level difference in voluntary diaphragm activation, although subtle differences cannot be excluded given the variability and modest sample size.

Sniff Pdi showed significant variation across the dyspnea subgroups, with much lower values observed in individuals with severe dyspnea (**Table 1**, overall ANOVA p-value = 0.03). Similarly, Mueller Pdi differed across dyspnea subgroups, with higher values in the no/mild dyspnea subgroup than in the moderate dyspnea subgroup (**Table 1**, overall ANOVA p-value = 0.05). No relevant differences were observed in sniff nasal pressure (**Table 1**). No significant differences in neural control (DVAI) were observed between patients and controls or among dyspnea subgroups (**Table 1**).

### Diaphragm ultrasound

Diaphragm ultrasound analysis demonstrated possible structural alterations in post-COVID individuals. Diaphragm thickening ratio did not differ statistically between the post COVID cohort and healthy controls (p=0.07).

In contrast, absolute diaphragm thickness was significantly reduced in post-COVID patients both at FRC and TLC compared to controls (p=0.02 and p<0.001 respectively, **Table 1**). No significant statistical differences were observed in any ultrasound values between subgroups based on dyspnea severity (**Table 1**).

## Discussion

In this exploratory study of non-hospitalized individuals with long COVID and persistent dyspnea, we observed lower TwPdi and lower right hemidiaphragm thickness compared to controls. These findings extend our previous observations in patients hospitalized for severe COVID-19 (Regmi et al., 2023; Spiesshoefer et al., 2024) and suggest that respiratory muscle involvement is not limited to individuals requiring hospitalization during the acute phase of infection. In line with our previous works (Regmi et al., 2023; Spiesshoefer et al., 2024), diaphragm impairment appears to persist well beyond the acute phase of the infection and may represent a key contributor to long-term symptom burden. The observed reduced diaphragm thickness at FRC and TLC may reflect changes in diaphragm structure. Although DTR was numerically lower in post-COVID participants, this difference was not statistically significant in the present cohort. Some routine physiological variables, such as capillary pO2, showed mild variation across the dyspnea subgroups; however, these findings were not sufficient to explain the observed symptom burden at the group level. Similarly, we observed reduced DLCO in some participants, consistent with previous studies (Mendez et al., 2021; Kang et al., 2024). Specifically, DLCO was below the LLN in nine participants, although none had a DLCO <60% predicted, and exploratory analyses showed no relevant associations between DLCO and dyspnea severity.

Given the absence of major cardiopulmonary abnormalities, diaphragm muscle weakness is a possible contributor for otherwise unexplained exertional dyspnea. This is consistent with findings from other cohorts, such as those with neuromuscular diseases, where exertional dyspnea is likely attributable to underlying respiratory muscle weakness (Henke et al., 2019; Spiesshoefer et al., 2019b). Respiratory muscle assessment may therefore complement standard cardiopulmonary testing in patients with otherwise unexplained exertional dyspnea. Furthermore, despite the differences in perceived dyspnea, 6MWD did not differ significantly across subgroups. This discrepancy suggests that distance-based exercise testing may not fully capture the mechanisms underlying exertional symptom perception and highlights the necessity of extended diagnostic workup.

The mechanisms underlying diaphragm impairment after SARS-CoV-2 infection remain incompletely understood but are likely multifactorial. Direct viral invasion of muscle tissue, although debated, may contribute to structural damage through local inflammation; postmortem studies have also shown viral infiltration or immunomodulatory changes of the diaphragm with development of fibrosis after infection (Shi et al., 2021). In addition, systemic inflammation and immune dysregulation during and after infection may lead to persistent myopathic changes or mitochondrial dysfunction, impairing muscle energy metabolism and contractility (Dres et al., 2017; Wang et al., 2022; Madsen et al., 2024). Another possible contributor is small fiber neuropathy or phrenic nerve involvement, which could subtly affect neuromuscular transmission (Law et al., 2022). Reduced physical activity may also promote deconditioning and atrophy, particularly in individuals with prolonged fatigue.

However, for previously hospitalized individuals, several confounding factors might at least partly contribute to respiratory muscle weakness (e.g. ICU bedding with critical illness-associated diaphragm dysfunction, steroid or antiviral medication use, or pre-existing conditions) (Dres et al., 2017). These hospitalization-related confouders were absent in the present cohort. However, our study design does not allow us to distinguish whether our findings are attributable to virus-related mechanisms or to post-infection mechanisms, such as reduced physical activity resulting from symptom burden.

The impact of respiratory muscle weakness in people with long COVID and otherwise unexplained exertional dyspnea who had initial mild disease is relevant for two reasons. Firstly, the proportion of patients with an initially mild, rather than severe, course of COVID-19 has increased due to high vaccination rates and emergence of less virulent strains of SARS-CoV-2. Secondly, we have shown that IMT is a potentially effective treatment option for exertional dyspnea in long COVID (Spiesshoefer et al., 2024).

Persistent dyspnea in long COVID is likely multifactorial. In addition to respiratory muscle involvement, other mechanisms reported in long-COVID cohorts include dysfunctional breathing, hyperventilation tendencies, autonomic dysregulation, impaired peripheral oxygen utilization and generalized deconditioning (Fresard et al., 2022; Astin et al., 2023). In our cohort, fatigue was closely associated with symptom burden. The strong association between exertional dyspnea and fatigue may coexist and interact with reduced physical activity and deconditioning, rather than representing entirely independent processes.

Several limitations of our exploratory study should be acknowledged. The inclusion of a highly selected patient population without underlying cardiac or pulmonary disease reduces the generalizability of this study to the normal population. Because this was a cross-sectional study without pre-COVID physiological measurements, we cannot determine whether the observed differences were pre-existing or developed during/after SARS-CoV-2 infection. Additionally, given the nature of our study, we cannot determine whether our findings are specific to COVID-19 or are generally post-infectious in nature. Furthermore, we did not observe a significant difference in 6MWT. Given that comprehensive cardiopulmonary exercise testing was not performed, and considering the known limitations of the 6MWT in detecting subtle functional impairments, the external validity of these findings may be limited. Finally, the relatively small sample size may limit statistical power, particularly for subgroup analyses and needs confirmation in larger, multicenter cohorts. Representative diaphragm ultrasound was performed on the right hemidiaphragm only, as previously described. Left hemidiaphragm was not assessed, potentially missing unilateral or asymmetrical diaphragm abnormalities. Another important limitation is the absence of a post-COVID comparison group without persistent dyspnea. Therefore, our design does not allow us to determine whether the observed diaphragm abnormalities are specific to symptomatic long COVID or may also be present in individuals recovering from COVID-infection without persistent dyspnea. Given the modest sample sizes and exploratory nature of several analyses, these findings and between group-comparisons should be interpreted cautiously. Formal blinding was not implemented for the respiratory muscle analysis, which may have introduced measurement bias.

Nevertheless, the persisting symptom burden in these individuals remains high and clinically relevant. These findings have clinical implications for both diagnosis and management. Standard cardiopulmonary evaluations may fail to detect respiratory muscle impairment, potentially leading to underdiagnosis of a treatable contributor. Importantly, it may be improvable by targeted therapeutic interventions such as IMT that directly addresses respiratory muscle weakness and have shown promising results in recent studies (Spiesshoefer et al., 2024; Correa et al., 2025).

## Conclusion

Diaphragm muscle impairment may contribute to otherwise unexplained exertional dyspnea after mild COVID-19, extending previous observations in hospitalized patients to a non-hospitalized population. Given the worldwide prevalence of long COVID, our exploratory findings suggest a distinct long COVID phenotype with exertional dyspnea related to diaphragm muscle impairment, and suggest the possibility for therapeutic interventions targeting this mechanism in this subgroup.

## Data Availability

The data that support the findings of this study are available from the corresponding author upon reasonable request.
